# Correction: Elevating CLIC4 in Multiple Cell Types Reveals a TGF-β Dependent Induction of a Dominant Negative Smad7 Splice Variant

**DOI:** 10.1371/journal.pone.0168629

**Published:** 2016-12-12

**Authors:** The PLOS ONE Staff

## Notice of Republication

This article was republished on November 18, 2016, to correct an error in the title that was introduced during the typesetting process. The publisher apologizes for the error. Please download this article again to view the correct version. The originally published, uncorrected article and the republished, corrected articles are provided here for reference.

## Supporting Information

S1 File Originally published, uncorrected article. (PDF)

S2 File Republished, corrected article. (PDF)
