# Supplementary material for: Elevating CLIC4 in Multiple Cell Types Reveals a TGF-β Dependent Induction of a Dominant Negative Smad7 Splice Variant
Source: PLoS One. 2016 Aug 18;11(8):e0161410. doi: 10.1371/journal.pone.0161410 (PMC4990216; doi:10.1371/journal.pone.0161410)
Supplement: S1 Fig — (A) Mouse embryonic fibroblasts and (B) Human Osteosarcoma cell line HOS. Red arrows indicate the split tags. Since our RNA-seq sequencing depth is too large to be displayed completely, we have truncated the browser view. (PPT) [file pone.0161410.s001.ppt]

## Slide 1
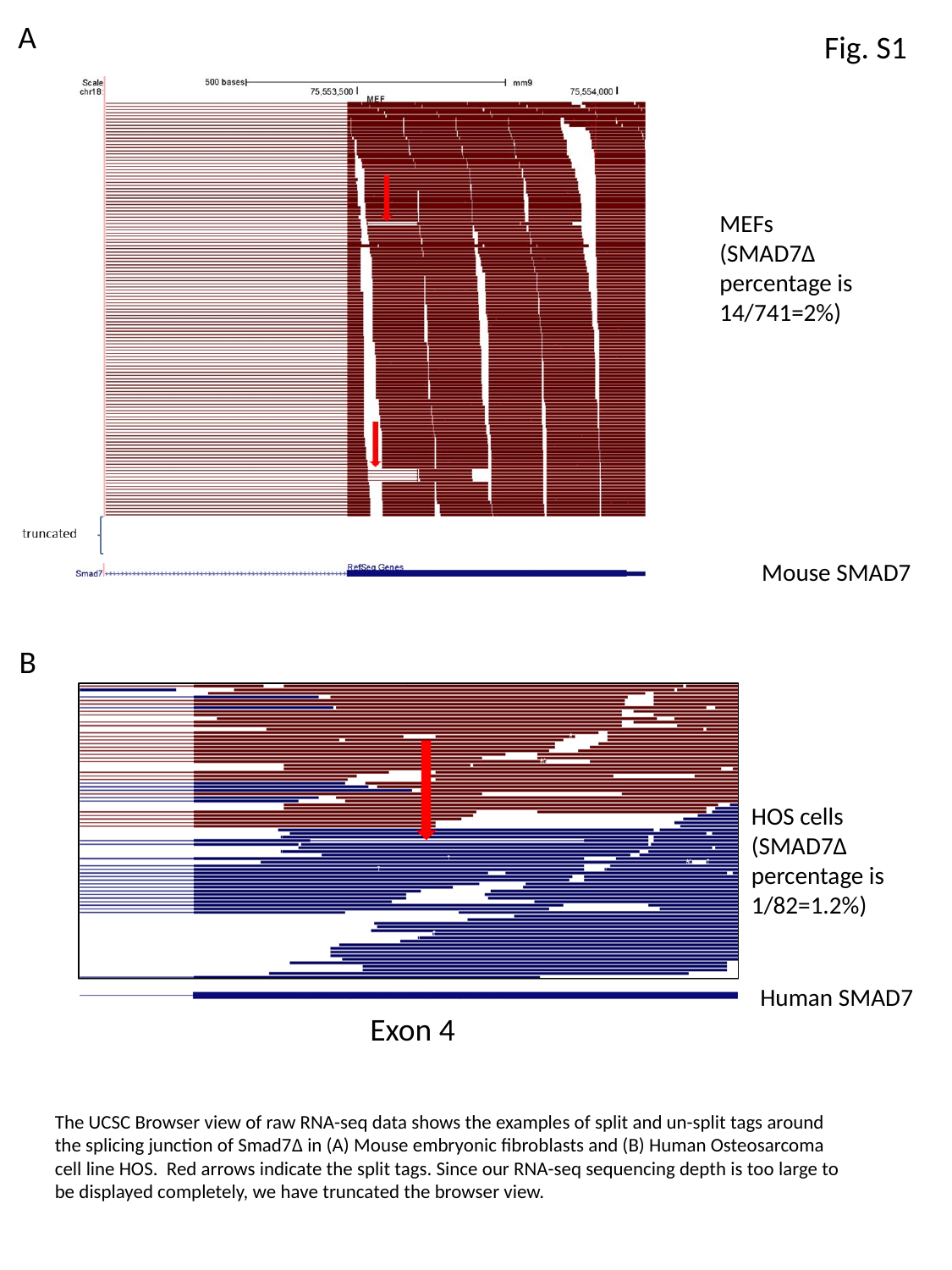

A
Fig. S1
MEFs
(SMAD7∆ percentage is 14/741=2%)
Mouse SMAD7
B
HOS cells (SMAD7∆ percentage is 1/82=1.2%)
Human SMAD7
Exon 4
The UCSC Browser view of raw RNA-seq data shows the examples of split and un-split tags around the splicing junction of Smad7Δ in (A) Mouse embryonic fibroblasts and (B) Human Osteosarcoma cell line HOS. Red arrows indicate the split tags. Since our RNA-seq sequencing depth is too large to be displayed completely, we have truncated the browser view.
