# Supplementary material for: Elevating CLIC4 in Multiple Cell Types Reveals a TGF-β Dependent Induction of a Dominant Negative Smad7 Splice Variant
Source: PLoS One. 2016 Aug 18;11(8):e0161410. doi: 10.1371/journal.pone.0161410 (PMC4990216; doi:10.1371/journal.pone.0161410)
Supplement: S2 Fig — (A) Nucleotide sequence spanning the alternative splice site of the human SMAD7 gene. Blue nucleotides are splicing sites. Red and blue nucleotides are skipped in SMAD7Δ. (B) Protein sequence spanning the alternative splice site of human Smad7 gene. Black amino acids are in SMAD7 while red amino acids are in SMAD7Δ. *, stop codon. (PPT) [file pone.0161410.s002.ppt]

## Slide 1
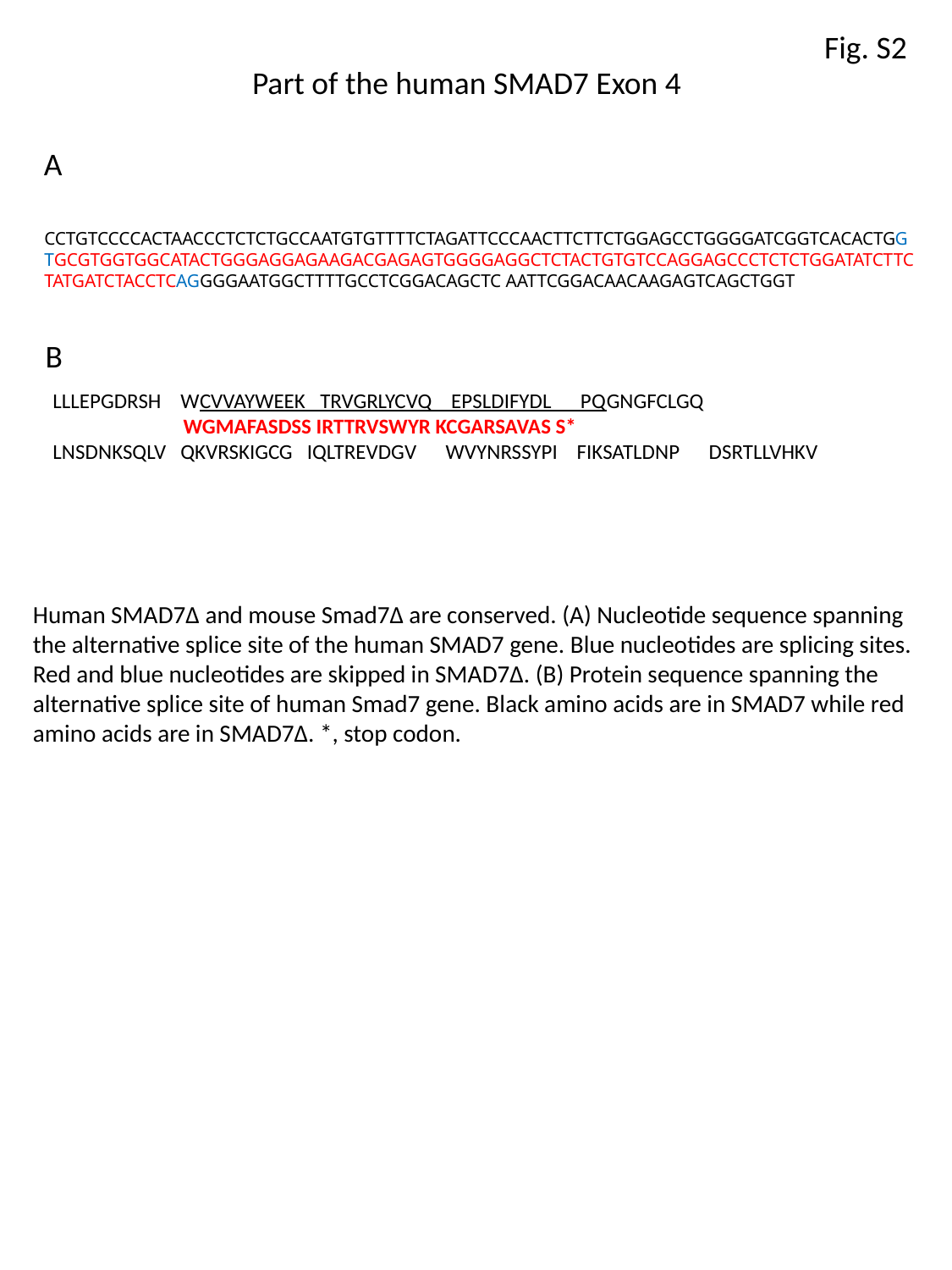

Fig. S2
Part of the human SMAD7 Exon 4
A
CCTGTCCCCACTAACCCTCTCTGCCAATGTGTTTTCTAGATTCCCAACTTCTTCTGGAGCCTGGGGATCGGTCACACTGGTGCGTGGTGGCATACTGGGAGGAGAAGACGAGAGTGGGGAGGCTCTACTGTGTCCAGGAGCCCTCTCTGGATATCTTCTATGATCTACCTCAGGGGAATGGCTTTTGCCTCGGACAGCTC AATTCGGACAACAAGAGTCAGCTGGT
B
LLLEPGDRSH WCVVAYWEEK TRVGRLYCVQ EPSLDIFYDL PQGNGFCLGQ
	 WGMAFASDSS IRTTRVSWYR KCGARSAVAS S*
LNSDNKSQLV QKVRSKIGCG IQLTREVDGV WVYNRSSYPI FIKSATLDNP DSRTLLVHKV
Human SMAD7∆ and mouse Smad7∆ are conserved. (A) Nucleotide sequence spanning the alternative splice site of the human SMAD7 gene. Blue nucleotides are splicing sites. Red and blue nucleotides are skipped in SMAD7∆. (B) Protein sequence spanning the alternative splice site of human Smad7 gene. Black amino acids are in SMAD7 while red amino acids are in SMAD7∆. *, stop codon.
