# Supplementary material for: Correction: Elevating CLIC4 in Multiple Cell Types Reveals a TGF-β Dependent Induction of a Dominant Negative Smad7 Splice Variant
Source: PLoS One. 2016 Dec 12;11(12):e0168629. doi: 10.1371/journal.pone.0168629 (PMC5153298; doi:10.1371/journal.pone.0168629)
Supplement: S2 File — (PDF) [file pone.0168629.s002.pdf]

PLOS | ONE

RESEARCH ARTICLE

Elevating CLIC4 in Multiple Cell Types Reveals a TGF-β Dependent Induction of a Dominant Negative Smad7 Splice Variant

Anjali Shukla, Yihan Yang, Sara Madanikia, Yan Ho, Mangmang Li, Vanesa Sanchez, Christophe Cataisson, Jing Huang, Stuart H. Yuspa*

Laboratory of Cancer Biology and Genetics, Center for Cancer Research, National Cancer Institute, Bethesda, Maryland, United States of America

* yuspas@mail.nih.gov

CrossMark
click for updates

OPEN ACCESS

Citation: Shukla A, Yang Y, Madanikia S, Ho Y, Li M, Sanchez V, et al. (2016) Elevating CLIC4 in Multiple Cell Types Reveals a TGF-β Dependent Induction of a Dominant Negative Smad7 Splice Variant. PLoS ONE 11(8): e0161410. doi:10.1371/journal.pone.0161410

Editor: Richard L. Eckert, University of Maryland School of Medicine, UNITED STATES

Received: May 25, 2016

Accepted: August 4, 2016

Published: August 18, 2016

Copyright: This is an open access article, free of all copyright, and may be freely reproduced, distributed, transmitted, modified, built upon, or otherwise used by anyone for any lawful purpose. The work is made available under the Creative Commons CC0 public domain dedication.

Data Availability Statement: All relevant data are within the paper and its Supporting Information files.

Funding: This work was conducted with funding to SHY from the NCI, CCR intramural program under project BC005445-30.

Competing Interests: The authors have declared that no competing interests exist.

Abstract

CLIC4 (Chloride intracellular channel 4) belongs to a family of putative intracellular chloride channel proteins expressed ubiquitously in multiple tissues. CLIC4 is predominantly soluble and traffics between the cytoplasm and nucleus and participates in cell cycle control and differentiation. Transforming growth factor beta (TGF-β) elevates CLIC4, which enhances TGF-β signaling through CLIC4 mediated stabilization of phospho-Smad2/3. CLIC4 is essential for TGF-β induced conversion of fibroblasts to myofibroblasts and expression of matrix proteins, signaling via the p38MAPK pathway. Therefore, regulation of TGF-β signaling is a major mechanism by which CLIC4 modifies normal growth and differentiation. We now report that elevated CLIC4 alters Smad7 function, a feedback inhibitor of the TGF-β pathway. Overexpression of CLIC4 in keratinocytes, mouse embryonic fibroblasts and other mouse and human cell types increases the expression of Smad7Δ, a novel truncated form of Smad7. The alternatively spliced Smad7Δ variant is missing 94bp in exon 4 of Smad 7 and is conserved between mouse and human cells. The deletion is predicted to lack the TGF-β signaling inhibitory MH2 domain of Smad7. Treatment with exogenous TGF-β1 also enhances expression of Smad7Δ that is amplified in the presence of CLIC4. While Smad7 expression inhibits TGF-β signaling, exogenously expressed Smad7Δ does not inhibit TGF-β signaling as determined by TGF-β dependent proliferation, reporter assays and phosphorylation of Smad proteins. Instead, exogenous Smad7Δ acts as a dominant negative inhibitor of Smad7, thus increasing TGF-β signaling. This discovery adds another dimension to the myriad ways by which CLIC4 modifies TGF-β signaling.

Introduction

The TGF-β signaling pathway importantly regulates numerous cellular activities including growth, differentiation, apoptosis, adhesion and motility [1]. These myriad effects are elicited by a signaling cascade from the cell membrane to the nucleus. The canonical TGF-β signaling

cascade is initiated when a TGF-β ligand binds to the TGF-β Type II receptor on the cell surface, and subsequently heterodimerizes with and phosphorylates the TGF-β Type I receptor [2]. This then leads to phosphorylation of Receptor Smad molecules (R-Smads), their association with Smad4 (Co-Smad) and translocation to the nucleus to regulate transcription of target genes. TGF-β signaling is fine tuned and regulated through multiple means, one of which is induction of expression of Inhibitory Smads (I-Smads) [2]. The expression and nuclear export of the I-Smads, Smad6 and Smad7, is induced by TGF-β signaling. Smad6 preferentially inhibits BMP signaling [3,4] whereas Smad7 inhibits both TGF-β and BMP signaling [5]. They inhibit Smad signaling by several mechanisms: Smad7 interferes with interaction of R-Smads with type I receptors and thus preventing R-Smad phosphorylation and activation [5]; Smad6 and Smad7 prevent complex formation between R-Smad and co-Smads that prevents R-Smad nuclear translocation and subsequent transcriptional regulation [5,6]; they interfere with functional Smad-DNA complex formation in the nucleus [7]; or directly regulate transcription in the nucleus [8].

CLIC4 (Chloride Intracellular Channel 4) is a highly conserved multifunctional member of a family of six proteins that are similar in size, highly homologous and participate in many signaling activities [9,10]. Of all family members, CLIC4 has been most extensively studied. The 28kD ubiquitously expressed CLIC4 is dimorphic, found both in intracellular membranes as well in soluble form in the cytoplasm and regulated by cellular redox state [11,12]. A major site of action of soluble CLIC4 appears to be in the nucleus where it modifies TGF-β signaling [13]. CLIC4 translocates from the cytoplasm to the nucleus under cellular stress including metabolic stress, growth arrest, apoptosis and DNA damage [14]. In the nucleus, CLIC4 enhances TGF-β signaling by associating with phosphorylated R-Smads and inhibiting their dephosphorylation by specific Smad phosphatase PPM1a, thereby prolonging the activated state of Smads and hence the TGF-β signal [13]. CLIC4 deficient mice display spontaneous skin erosions and delayed wound healing possibly through altered TGF-β signaling [15]. During carcinogenesis, CLIC4 mirrors the dual nature of TGF-β in causing context dependent tumor suppression or enhancement. Elevating CLIC4 levels in tumor epithelium suppresses tumor growth that is coincident with enhanced TGF-β signaling [16]. In contrast, elevating CLIC4 in tumor stroma via TGF-β and p38 signaling enhances tumor growth and tumor cell invasion [17]. Here we report that CLIC4 has devised another strategy to enhance TGF-β signaling: by inducing expression of a novel, alternate form of Smad7 in collaboration with TGF-β. We describe the identification of this previously uncharacterized shorter form of Smad7 that appears to be the result of an alternative splicing event and designate it as Smad7Δ. **The splicing event as well as sequences of Smad7Δ are conserved between mouse and human cell types**. Expression of endogenous Smad7Δ is induced upon overexpression of CLIC4 and by TGF-β treatment of keratinocytes and other cell types in the presence of CLIC4. Exogenous Smad7Δ acts as a dominant inhibitor of Smad7 and enhances TGF-β signaling.

Results

Expression of exogenous CLIC4 induces a shorter form of Smad7

Overexpressing CLIC4 in mouse keratinocytes revealed two Smad7 transcripts: the expected amplicon and a transcript producing an approximately 100bp shorter band (Fig 1A). Time course analysis indicated that the shorter band only appears after CLIC4 is elevated substantially by adenoviral transduction and is not related to adenoviral infection alone (Fig 1B). Sequence analysis of this shorter band reveals the absence of 94bp from exon 4 of Smad7 (Fig 1C, blue line). This novel form of Smad7 was termed Smad7Δ. Primers specific to Smad7Δ were designed (see experimental procedures and Fig 1D) and used for quantitative real time

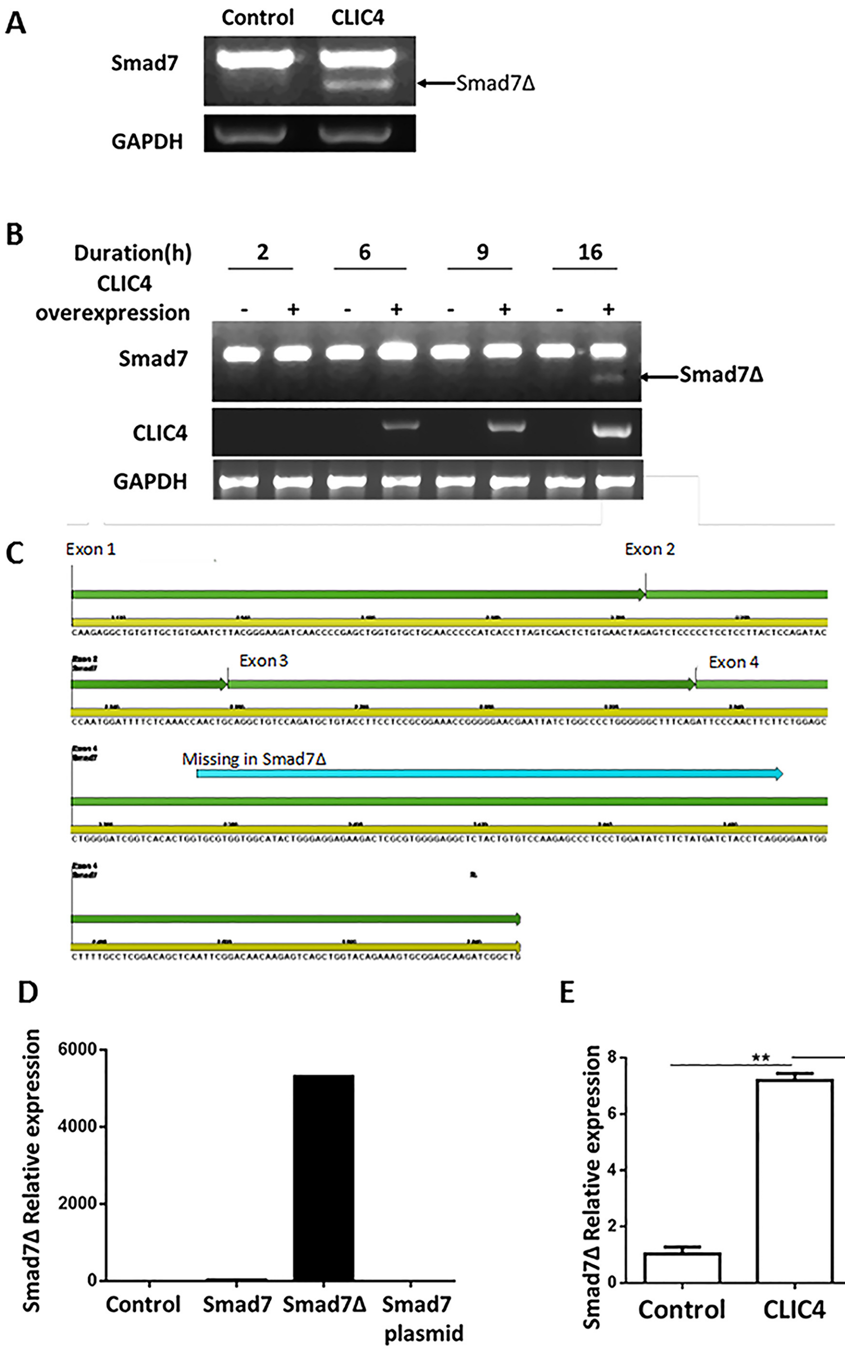

Fig 1. CLIC4 overexpression induces expression of Smad7Δ. A. Primary keratinocytes transduced with Vector (Control) or CLIC4 expressing adenoviruses for 16h were subjected to RT-PCR using Smad7 primers. B. Primary keratinocytes transduced with Vector (Control) or CLIC4 expressing adenoviruses for indicated periods of time were analyzed for Smad7 and CLIC4 by RT-PCR. GAPDH was used as control for A and B. C. Genomic organization of Smad7 gene showing the region deleted in SmadΔ in blue, based on sequencing analysis. D. To validate Smad7Δ specific primers, real time PCR was carried out on reverse transcribed RNA from control adenovirus, Smad7 or Smad7Δ adenovirus transduced keratinocytes or Smad7 plasmid DNA. Results are expressed relative to control sample which is assigned an arbitrary value of 1.0. E. Real time PCR for Smad7Δ was carried out on samples from Vector (Control), CLIC4 or GFP transduced keratinocytes and presented here as relative to control sample. Statistical comparisons were performed as indicated. **p<0.005.

doi:10.1371/journal.pone.0161410.g001

PCR revealing that overexpression of CLIC4 induces expression of Smad7Δ while the adeno-vector and adeno-GFP do not (Fig 1E).

TGF-β treatment enhances expression of Smad7Δ in a CLIC4 dependent manner

We have previously shown that CLIC4 enhances TGF-β signaling. In order to determine if the induction of Smad7Δ by CLIC4 is TGF-β dependent, we blocked TGF-β signaling using the ALK5 receptor blocker SB431542. At this treatment dose, the extent of Smad7Δ induction achieved by CLIC4 is reduced (Fig 2A) but not completely abrogated. The involvement of TGF-β in the induction of Smad7Δ is further shown in Fig 2B where TGF-β treatment of keratinocytes for 2 hours induces expression of Smad7Δ. Induction of Smad7Δ by CLIC4 overexpression as well as by TGF-β treatment or the combination was also noted in several other mouse and human cell types (Fig 2C) indicating this is an across species response. A cooperative interaction between CLIC4 and TGF-β in Smad7Δ generation is shown further in Fig 2D. Primary keratinocytes from CLIC4 KO mice when treated with various doses of TGF-β1 for 2h failed to upregulate Smad7Δ expression to a level comparable to WT keratinocytes as detected by q-PCR.

Sequence analysis of Smad7Δ

Fig 3A shows partial exon 4 cDNA sequence of Smad7 with the deleted region in Smad7Δ indicated in red. Smad7 cDNA sequence with this deletion translates into a truncated protein Smad7Δ that terminates from a frame shift and has a novel C terminus highlighted in red in Fig 3B. The N terminus of Smad7Δ is predicted to be identical to that of Smad7. Based on this information, adenoviral expression vectors for Smad7 and Smad7Δ were constructed. Immunoblot analysis of primary mouse keratinocytes transduced with Smad7 and Smad7Δ expressing adenoviruses probed with an N-terminal Smad7 antibody confirmed that these constructs overexpressed proteins of expected sizes of 45kD and 31kD respectively (Fig 3C).

Smad7Δ acts as a dominant inhibitor of Smad7

Using the adenoviral vectors we were able to test if Smad7Δ affects TGF-β dependent transcription (Fig 4A). As expected, increasing expression of Smad7 decreases p3TP luciferase TGF-β reporter activity. The same assay reveals that transduction with increasing amount of Smad7Δ expressing adenovirus increases TGF-β reporter activity. A combination of Smad7 and Smad7Δ increases reporter activity irrespective of proportion of the two viruses. For example, 2.5 MOI of Smad7Δ prevents repression of TGF-β reporter activity even by 10 MOI of Smad7 virus. This shows that exogenous Smad7Δ functions as a dominant inhibitor of Smad7 function to repress TGF-β signaling. Support for this conclusion also comes from a study of adenovirally transduced keratinocytes analyzed for the expression of various TGF-β downstream genes on a TGF-β PCR expression array. Following a 1h treatment with TGF-β, exogenous Smad7 inhibits or enhances expression of various TGF-β downstream genes whereas Smad7Δ does the opposite (Fig 4B). Co-expression of Smad7 and Smad7Δ resulted in TGF-β signaling changes similar to Smad7Δ alone supporting the indication that Smad7Δ functions in a dominant negative manner over Smad7. Hprt1 and Hsp90ab1 are used as house keeping genes.

Since Smad7 primarily inhibits Smad signaling, we examined the effect of Smad7, Smad7Δ and their co-expression on Smad2 phosphorylation. In TGF-β treated keratinocytes, Smad7 inhibits phosphorylation of Smad2 but Smad7Δ actually enhances it. Interestingly keratinocytes transduced with a combination of Smad7 and Smad7Δ have a higher amount of phospho-Smad2 than Smad7 alone transduced cells (Fig 4C).

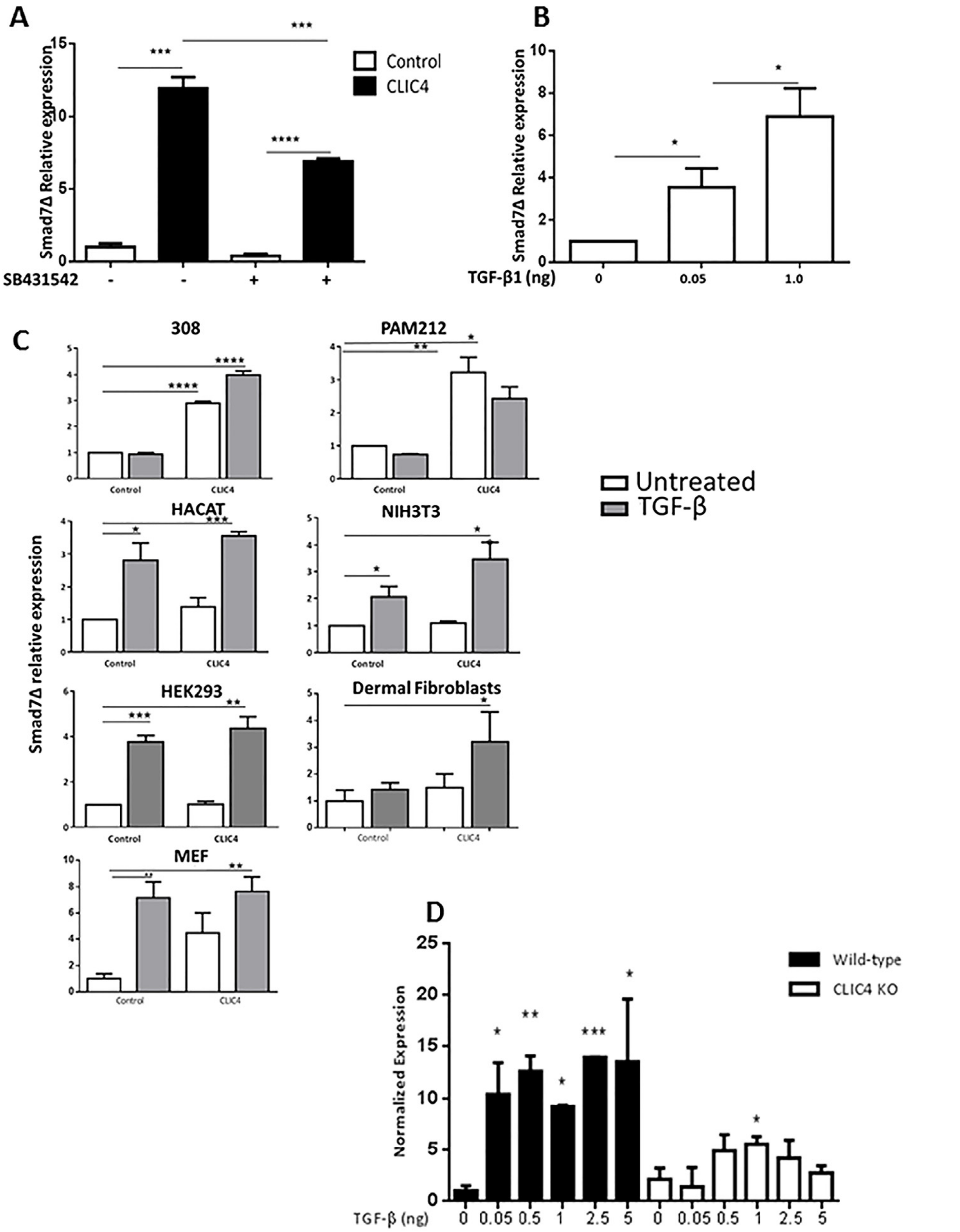

Fig 2. TGF-β induces expression of Smad7Δ. A. Real time PCR for Smad7Δ was carried out on primary keratinocytes transduced with Vector (Control) or CLIC4 expressing adenoviruses +/- 30 min pretreatment with 5μM ALK5 blocker SB431542. B. Primary keratinocytes treated with indicated doses of TGF-β for 2h in serum free media were analyzed for Smad7Δ by real time PCR. C. Indicated cell types were transduced with empty vector (Control) or CLIC4 expressing adenovirus and subsequently treated or not with 1ng/ml TGF-β1 for 2h. Total RNA was reverse transcribed and analyzed for Smad7Δ expression by q-pcr. D. Primary keratinocytes from WT or CLIC4 KO mice treated with indicated doses of TGF- β1 for 2h in serum free media were analyzed for Smad7Δ by real time PCR. Statistical analysis compared each treated sample with its respective untreated control. A,B,C,D. Smad7Δ levels were normalized to GAPDH content and are presented as relative to control untreated sample. A,B,C,D. Statistical comparisons were carried out as indicated. *p<0.05, **p<0.005, ***p<0.0005.

doi:10.1371/journal.pone.0161410.g002

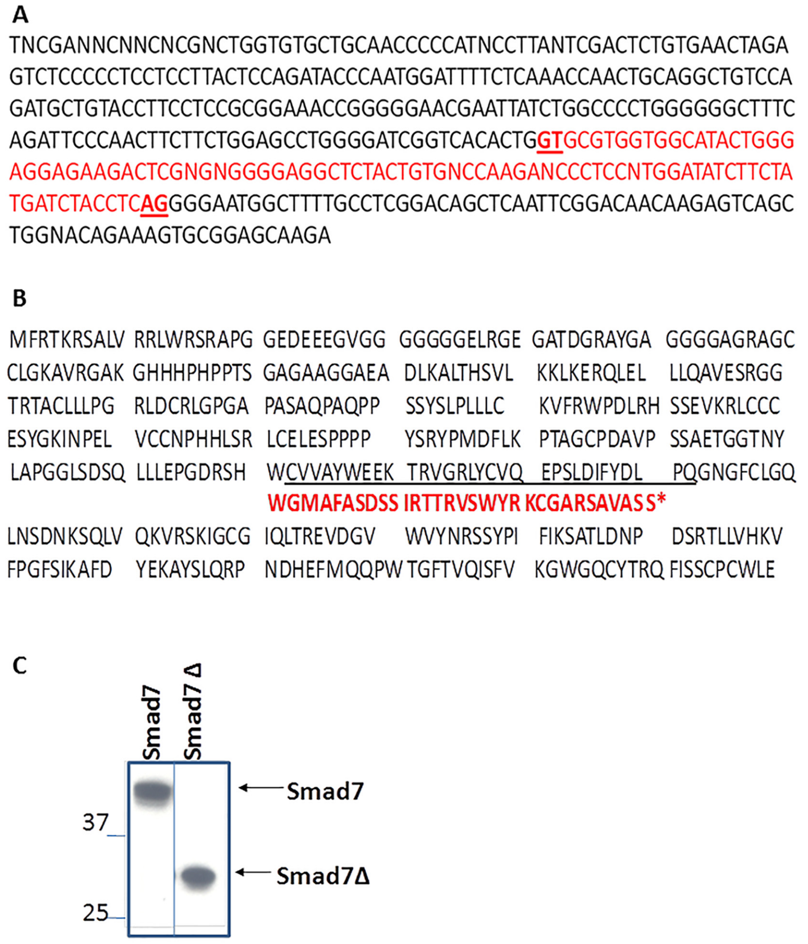

Fig 3. cDNA and predicted protein sequence of Smad7Δ. A. cDNA sequence of part of exon 4 of Smad7 showing the region missing in Smad7Δ in red. B. Protein sequence of Smad7 predicted upon *in silico* translation of Smad7 cDNA sequence. In red is the predicted C terminus amino acid sequence of Smad7Δ. C. Primary keratinocytes were transduced with adenoviruses expressing Smad7 or Smad7Δ and immunoblotted using an N-terminal Smad7 antibody. Bands appear at expected sizes of 45 and 31kD for Smad7 and Smad7Δ respectively.Smad7Δ transduced cells also show Smad7 protein at higher exposure, not shown here.

doi:10.1371/journal.pone.0161410.g003

Growth inhibition is a hallmark of active TGF-β signaling in epithelial cells [18, 19]. In a thymidine incorporation assay, while TGF-β treatment decreased proliferation of keratinocytes, exogenous Smad7 abrogated this inhibition as expected (Fig 4D). Neither transduced Smad7Δ expression alone nor a combination of transduced Smad7 and Smad7Δ inhibited the effect of TGF-β, again showing that Smad7Δ had the capacity to over-ride the effect of Smad7 on TGF-β dependent growth inhibition.

Smad7Δ is expressed constitutively in mouse and human cells, is highly inducible and associated with pathology

We used RNA-seq coupled to the TopHat algorithm in MEF cells and human osteosarcoma cell line HOS as an un-biased approach to analyze splicing junctions [20]. In the absence of entered *a priori* knowledge, the program detected the presence of split tags that spanned the predicted splicing junction for Smad7Δ (S1 Fig), therefore demonstrating that the splicing

A

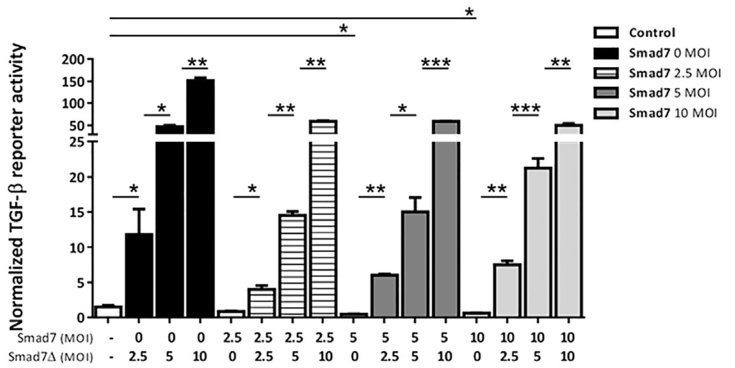

B

Fold change over vector control expression			
Gene	Smad7 wt	Smad7Δ	Smad7 wt + Δ
Bmp6	1.02	3.12	4.85
Cd79a	0.47	3.84	2.69
Col1a1	0.70	1.91	1.32
Gdf1	0.70	1.54	1.47
Gsc	10.82	1.42	2.33
IL6	0.36	2.30	1.08
Itgb7	34.66	1.86	4.16
Junb	33.01	1.87	1.12
Lefty1	3.00	1.11	1.20
Ltbp1	0.05	1.27	1.33
Nbl1	9.09	2.93	1.88
Nr0b1	102.89	1.29	4.08
Serpine1	0.46	2.63	1.19
Tgfb1	0.54	2.30	1.24
Hprt1	1.47	1.43	1.60
Hsp90ab1	1.02	0.94	1.12

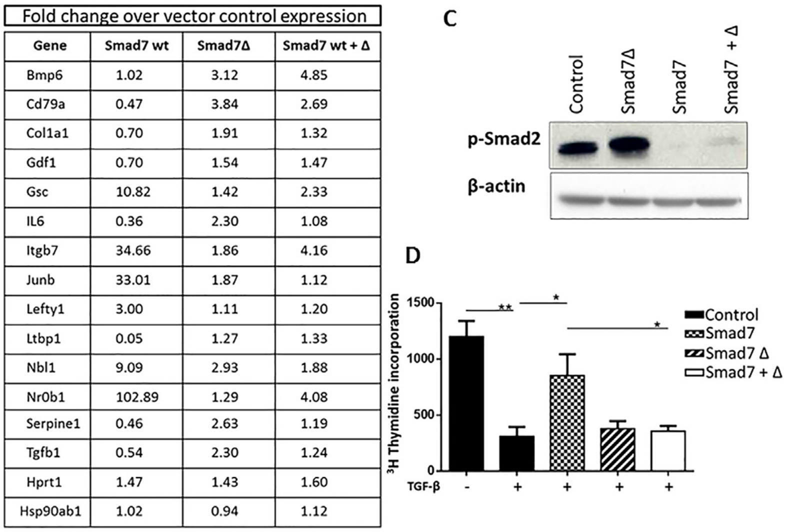

Fig 4. Smad7Δ functions as a dominant inhibitor of Smad7. A. Primary keratinocytes were transfected with p3TP luciferase and pRLTK plasmids for 24h, then transduced with Smad7 and Smad7Δ adenoviruses as indicated and treated with TGF-β for 16h. Luciferase assays were performed and represented here as fold change relative to that of Vector transduced control sample. B. Primary keratinocytes were transduced with Vector (Control), Smad7, Smad7Δ or a combination of both for 16h, subsequently treated with TGF-β1 for 1h and analyzed on a TGF-β signaling PCR array by real time PCR. Data are presented as fold change over control sample transduced with empty vector. C. Primary keratinocytes were transduced as in B, treated with TGF-β1 for 1h and immunoblotted for p-Smad2. β-actin was used as loading control. D. Primary keratinocytes were transduced as in B, treated with TGF-β for 16h and subsequently exposed to ^{3}H-thymidine for 3h. ^{3}H-Thymidine incorporation was determined by scintillation counting of lysates and displayed as raw counts normalized to cell number. A, D Statistical comparison was performed as indicated. *p<0.05, **p<0.005, ***p<0.0005.

doi:10.1371/journal.pone.0161410.g004

variant Smad7Δ exists constitutively in both mouse and human cells and is naturally occurring. Importantly, the nucleotide and protein sequence of Smad7Δ is conserved between mouse and human species (S2 Fig). In the unstimulated state, an analysis of split vs unsplit tags yielded a 2% total abundance of Smad7Δ transcripts relative to total Smad 7 transcripts in MEFs while HOS showed a relative abundance of 1.2%. (Fig 5A). Introduction of exogenous CLIC4 into MEFs modestly increased the expression of Smad7Δ that was inhibited by blocking the TGFBRII indicating it is TGF-β dependent. In contrast TGF-β treatment of MEFs at 1 ng/ml induced expression of Smad7Δ transcripts 20–30 fold suggesting it is a primary signal for regulating the splicing of the Smad7 transcript (Fig 5B and 5C). Interestingly, the level of induction

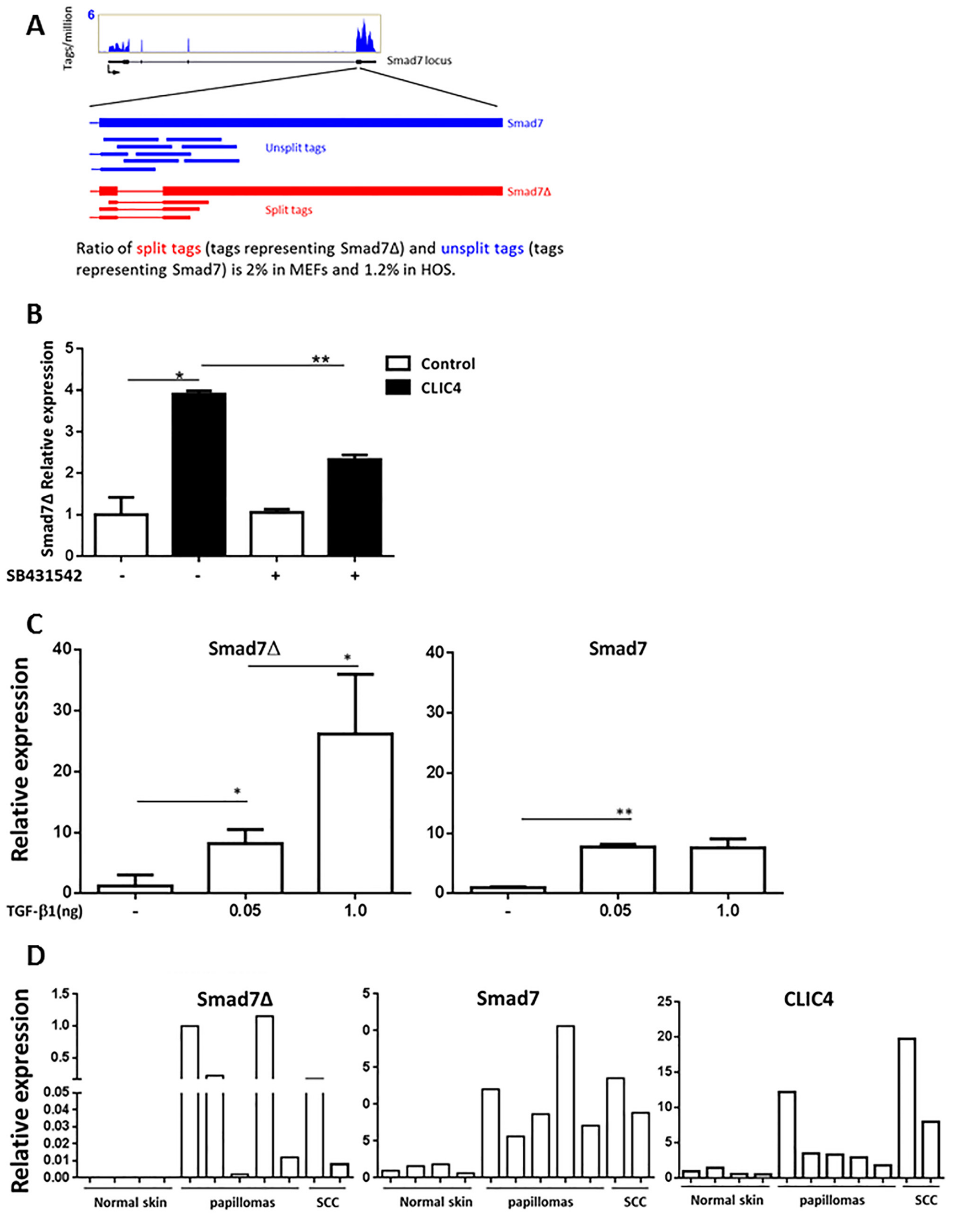

Fig 5. Smad7Δ is an alternately spliced isoform of Smad7, occurs constitutively, is highly inducible and associated with pathology. A. Shown is the genomic view of the Smad7 locus using RNAseq data in MEF and HOS cells. The spliced part of exon 4 of the Smad7 gene was enhanced to illustrate Smad7Δ and Smad7. Blue thick bar shows un-spliced Smad7 while red thick bar the Smad7Δ variant. Blue thin bars indicate un-split tags for the normal variant while red thin bars the split tags for Smad7Δ. The ratio of split tags and un-split tags is 2% in MEFs and 1.2% in HOS cells. Raw data are shown in S1B Fig. Primary MEFs were transduced with Vector (Control) or CLIC4 expressing adenoviruses with or without 30 min pretreatment with the 5μM ALK5 blocker SB431542. Smad7Δ expression was analyzed via real time PCR and is presented as fold change over untreated Vector only (Control) transduced MEFs. C. Primary MEFs were treated with 0.05 and 1ng TGF-β1 for 2h in serum free media and Smad7Δ and Smad7 levels analyzed by real time PCR. B,C, Statistical comparison was performed as indicated. *p<0.05, **p<0.005. D. Real time PCR analyses for Smad7Δ,Smad7 and CLIC4 were performed on RNA isolated from normal skin, squamous papillomas and squamous cell carcinomas arising from orthografts of oncogenic ras transduced primary mouse keratinocytes. Each bar represents tumor or skin from a separate mouse normalized to first papilloma for Smad7Δ and first normal skin sample for Smad7 and CLIC4.

doi:10.1371/journal.pone.0161410.g005

of Smad7 transcripts by TGF-β in these cells was substantially less than the induction of the Smad7Δ transcript implying that TGF-β preferentially elevates the variant transcript. As in many ying-yang functions of TGF-β, this preferential induction of the Δ transcript could serve as a mechanism to bring the two opposing protein functions into better balance during exposure. To determine if the Smad7Δ transcript is detected in vivo or altered in disease states, we examined RNA by real time PCR from normal skin and benign and malignant squamous tumors induced by oncogenic ras transduction (Fig 5D). Smad7Δ could not be detected in normal skin but low levels of Smad 7 were detectable. In contrast, Smad7Δ transcripts were readily detectable in all tumors at variable but highly elevated levels in some tumors, even in the benign state. Smad 7 transcripts were also elevated in both benign and malignant tumors where TGF-β levels are generally high [21]. Probing the same tumors for CLIC4 transcripts showed a similar pattern of variability with some tumors correlating high expression of both CLIC4 and Smad7Δ transcripts (Fig 5D). However, whether these transcripts are expressed in the same cells or same compartments of the tumor cannot be determined by these methods. Nevertheless, these findings suggest that the balance of these opposing and facilitating modifying factors for TGF-β signaling could influence tumor development.

Discussion

The complexity of TGF-β signaling mediators lends specificity to the wide array of actions and interactions that define this pathway. R-Smads consist of the MH1 (Mad-homology 1) and MH2 domains connected by a linker region [2]. Their C terminus has a SSXS motif that is phosphorylated by activated Type I receptor. The Co-Smads contain MH1 and MH2 domains but do not contain the SSXS motif at their C terminus and hence cannot be phosphorylated by the receptors. I-Smads have a conserved MH2 domain but also lack the SSXS motif. Their N-termini lack the DNA-binding domain present in MH1 domains of the R-Smads and Co-Smads. The C-terminal MH2 domains of Smad6 and Smad7 are responsible for their interaction with the Type I receptor and for inhibition of the TGF-β signaling pathway [22,23]. The MH2 domains of Smad6 and Smad7 are sufficient to repress TGF-β signaling while isolated N-terminal domains of these proteins are unable to cause this repression themselves. The N-domain of Smad7 determines its subcellular localization and aids the inhibitory activity of its MH2 domain through physical interaction with the MH2 domain [23]. As predicted in Fig 3B, the observed deletion of 94bp in exon 4 of Smad7 produces a frame shift and early truncation of the protein, eliminating the MH2 domain in Smad7Δ (Fig 6). In the absence of an MH2 domain and an altered C-terminus, it would be expected that Smad7Δ would be unable to inhibit TGF-β signaling. Our results show that Smad7Δ does not inhibit TGF-β signaling but instead acts as a dominant inhibitor of Smad7 function and potentiates TGF-β signaling.

Overexpression of CLIC4 in keratinocytes first revealed the existence of this variant and led to the understanding of its TGF-β inducibility. Examination of multiple cell types suggests the Smad7Δ transcript is widely expressed in both human and mouse cells albeit at low levels but inducible. As depicted by RNA-seq analysis of primary MEFs and osteosarcoma cell line HOS, Smad7Δ is expressed in baseline, unmanipulated conditions in normal primary cells as well as in disease state. qPCR indicates it is induced by TGF-β treatment in MEFs and multiple other cell types. Thus it seems likely that Smad7Δ participates in the regulation of TGF-β signaling as required, and can modify the downstream readout of this pathway. Our results show that CLIC4 is important for induction of Smad7Δ. The enhanced induction of Smad7Δ by combined TGF-β treatment and overexpression of CLIC4 supports this idea as do the reduction in TGF- β induced Smad7Δ transcript in CLIC4 KO keratinocytes and decrease in CLIC4-induced Smad7Δ transcripts when TGF-β signaling is blocked (Fig 2). That the Δ transcript had

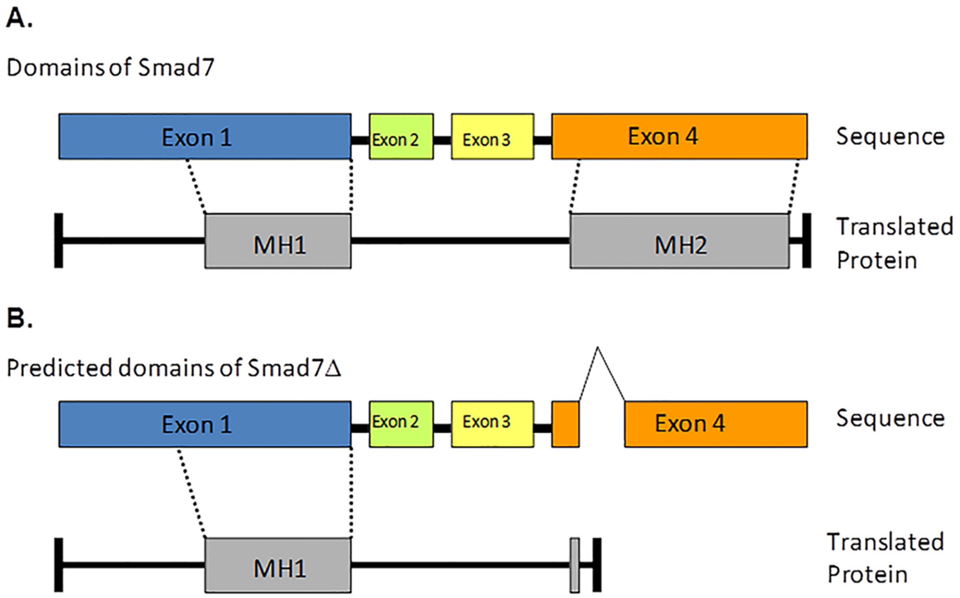

Fig 6. Comparison of Smad7 and Smad7Δ domains. A. Exon arrangement of Smad7 and location of MH1 and MH2 domains. B. Exon arrangement of Smad7Δ compared to that of Smad7 and the resulting MH1 and absence of MH2 domain predicted based on sequence analysis.

doi:10.1371/journal.pone.0161410.g006

not been seen previously in Smad7 studies suggests some selectivity, either direct or indirect, for a mechanism related to expression of CLIC4. It is likely that our previously reported mechanism where TGF-β enhances CLIC4 expression that in turn stimulates TGF-β signaling by sustaining the phosphorylated Smad2/3 signal is operative here as well [13]. The enhanced induction of Smad7Δ by combined TGF- β treatment and overexpression of CLIC4 supports this idea. Since CLIC4 is constitutive in most cell types and TGF-β is in the microenvironment of all tissues, it is likely they participate in basal regulation of Smad7Δ. Additional studies are required to interrogate this mechanism.

Analysis of the borders of the deletion that gives rise to Smad7Δ reveals a GT/AG motif (highlighted in bold red and underlined in Fig 3A) that is the hallmark of alternate splicing and denotes a splice donor and acceptor site. RNA-seq analysis of MEFs and human osteosarcoma cells further shows that Smad7Δ arises due to novel splicing and that we have uncovered an alternately spliced form of Smad7. TGF-β signaling has been shown to participate in splicing of many genes [24,25]. It is likely that alternate splicing of Smad7 to produce Smad7Δ involves enhanced TGF-β signaling brought about by CLIC4. According to an analysis using NCBI's Aceview program, transcription of the Smad7 gene could produce at least 7 different alternately spliced mRNAs all putatively encoding good proteins. There are 5 probable alternate promoters and 2 validated polyadenylation sites. The mRNAs appear to differ by truncation of the 5' end, overlapping exons with different boundaries, alternative splicing or retention of one intron. Smad proteins are commonly known to have several splice isoforms. A splice form of Smad2 that lacks exon 3 is differentially expressed during mouse brain development and aging [26]. Alternately spliced forms Smad5 and Smad5β diverge at the junction of exon 6, with Smad5β found to be more highly expressed in undifferentiated hematopoietic stem cells than in terminally differentiated peripheral blood leukocytes [27]. Three human Smad6 isoforms have been identified [28]. Apart from the full length Smad6 that consists of an N-terminal MH1 domain and a C-terminal MH2 domain, Smad6s exhibits a truncated MH1 domain

while Smad6B, due to use of an alternate exon 4, has a C-terminal truncation leading to lack of the entire MH2 domain and parts of the linker region. Smad8B, a splice variant of Smad8 lacks the C-terminal SXSS motif and inhibits Smad8 mediated signaling [29]. Smad8B acts as a dominant inhibitor of BMP signaling. Analysis of the annotated RNA database does not display Smad7Δ. Therefore this study discovers a constitutively expressed, hitherto unannotated form of Smad7 that functions at least experimentally as an inhibitor of Smad7 and potentiates TGF-β signaling.

Smad7 is frequently elevated in various tumor types including squamous cancers of the skin [30,31,32,33]. We now show that the alternatively spliced variant Smad7Δ is also increased in both benign and malignant tumors in vivo relative to normal skin where it can't be detected by our methods. Perhaps the Smad7Δ upregulation in skin tumors observed in our study is a mechanism to antagonize the impact of Smad7 increase and restore selective responses to TGF-β.

Due to the immense significance of TGF-β signaling in health and disease, the existence of Smad7Δ provides another mechanism to modify the activity of this pathway [34]. This discovery enriches our understanding of the vast and intricate world of TGF-β signaling that controls numerous biological functions via incompletely comprehended mechanisms. It also re-emphasizes the role of CLIC4 in modifying TGF-β signaling and indicates that CLIC4 may utilize multiple ways to do so.

Conclusions

This study reports an alternately-spliced form of Smad7, Smad7Δ, that is induced by TGF-β and CLIC4, is a dominant inhibitor of Smad7 and enhances TGF-β signaling. It thus reveals a previously undetected novel mechanism that fine-tunes TGF-β signaling.

Methods

Cell culture, expression vectors, pathway inhibitors and transfection

Primary keratinocytes from Balb/c newborn mice and newborn CLIC4 knockout (KO) mice on an FVB/N background backcrossed to FVB/N [15] and dermal fibroblasts from Balb/c newborn mice were obtained as per the specifications of protocol (#LCCTP-005) approved by the National Cancer Institute and NIH Animal Care and Use Committee and were prepared and cultured according to established methods [35]. MEFs were obtained from 13.5-day embryos of C57BL/6 background according to protocol (#LCBG-006) approved by the National Cancer Institute and NIH Animal Care and Use Committee and grown in DMEM+15% FBS using the procedures described previously [36]. All animals were maintained, physically assessed every day, fed ad libidum, bred and euthanized by CO2 inhalation at experimental endpoint as per their respective protocols. Keratinocyte cell lines 308 and PAM212 were cultured in Eagle's Minimum Essential medium supplemented with 0.05 mM $CaCl_2$ and 8% chelex treated fetal bovine serum. HACAT and HEK293 cells were cultured in DMEM. Null and CLIC4 expressing adenoviruses have been described before [14]. Smad7 and Smad7Δ expressing adenoviral constructs were engineered at the Protein Expression laboratory, NCI Frederick. SB431542 was obtained from Sigma- Aldrich.

Antibodies and immunoblotting

Phospho-Smad2 and total Smad2 antibodies were from Cell Signaling Technologies. Smad7 was detected using the N-19 Smad7 antibody from Santa Cruz Biotechnologies and β-actin was from Abcam. Protein expression was analyzed by immunoblotting. Briefly, cells were washed

and scraped into lysis buffer (Cell Signaling Technologies). 25 μg of protein was subjected to SDS-PAGE and immunoblotting and visualized using enhanced chemiluminescence (Pierce Biotechnology, Inc).

Real time PCR

Total RNA from cells was isolated using Trizol (Invitrogen), and reverse transcribed using SuperScript III First Strand kit (Invitrogen). SYBR Green (Biorad) based real-time PCR analysis was carried out using predesigned RT2 qPCR assay primers (Qiagen) for Smad7, CLIC4 and GAPDH, Bio-Rad iQ5 iCycler and Gene Expression Macro. Sequences for Smad7Δ primers are: Fw: TCTCCCCCTCCTCCTTACTC; Re: CAAAAGCCATTCCCCAGTGT. Smad7Δ expression was quantified using the SYBR Green based method as for other PCR analyses. Results are expressed as relative units after normalization to GAPDH expression levels. For TGF-β PCR array (Qiagen), cDNA from Balb/c keratinocytes transduced with Smad7, Smad7Δ or a 1:1 mix of Smad7 and Smad7Δ followed by TGF-β1 treatment for 1h was subjected to SYBR Green based real time PCR and analyzed using the Biorad Gene Expression Macro PCR analysis software.

Luciferase assay

Luciferase assay was carried out using TGF-β responsive p3TP luciferase reporter (p3TP-lux). Primary Balb/c keratinocytes, plated in 12-well culture plates, were transfected with p3TP-lux at a concentration of 2.0 μg/well. To control for transfection efficiency, 0.2 μg/well of pRLTK plasmid was co-transfected. 24h later, cells in quadruplicate were transduced with empty vector (Null), Smad7, Smad7Δ or a combination of Smad7 and Smad7Δ adenovirus at MOI as indicated in Fig 4A and co-treated with TGF-β1 (50 pg/ml) for 16h. Luciferase activity was determined in cell extracts using the Dual Luciferase Reporter assay system (Promega) and normalized to renilla luciferase activity.

^{3}H-Thymidine incorporation assay

To assay for DNA synthesis, keratinocytes were plated in 24-well plates and transduced with control (Null), Smad7, Smad7Δ or a 1:1 mix of Smad7 and Smad7Δ expressing adenoviruses for 16h. Some wells were co-treated with TGF-β1 (50 pg/ml). ^{3}H-Thymidine (1 μCi / well) was added to the wells for 3 hrs before the end of the treatment. Cells were fixed using methanol and acetic acid (in a 3:1 ratio), solubilized in 5N NaOH and incorporated counts measured using a scintillation counter and normalized to cell number.

RNA-seq

RNA was extracted from primary 13.5-day MEF cells and HOS (human osteosarcoma) cells using the GenElute Mammalian total RNA (Sigma). 1 μg of total RNA was sent to the CCR sequencing facility on a HiSeq 2000 platform. 100bp pair-end sequencing tags were analyzed using the TopHat algorithm to search for novel splicing junctions [20]. In house programs were used to make the genomic view of RNA-seq on the Smad7 locus and to calculate the ratio of split tags versus un-split tags. This ratio represents the amount of Smad7Δ versus Smad7.

Generation of tumors and extraction of tumor RNA

On day 3 in culture, primary C57BL/6NCr mouse keratinocytes were infected with the v-*ras*^{Ha} retrovirus and trypsinized and used for grafting on day 8 as described previously [35]. 4 million keratinocytes were mixed with 5 million SENCAR mouse primary dermal fibroblasts (cultured

for 1 wk) and grafted onto the back of nude mice on a prepared skin graft site located in the midback region. Mouse studies were performed under a protocol (ASP#LCCTP-053) approved by the National Cancer Institute (NCI) and NIH Animal Care and Use Committee under the specifications of which they were maintained, provided pellet food and water ad libidum, physically assessed every day and euthanized by CO_2 inhalation at experimental end point. Tumors were pulverized and RNA extracted using Trizol according to manufacturer's protocol (Invitrogen) and further purified through a Qiagen column with on-column DNA digest according to manufacturer protocol.

Statistics

All experiments were repeated a minimum of two times and data were subjected to unpaired t-test. P-values are indicated in figure legends. **** $p<0.0001$, *** $p<0.0005$, ** $p<0.005$, $^{*}p<0.05$.

Supporting Information

S1 Fig. The UCSC Browser view of raw RNA-seq data shows examples of split and un-split tags around the splicing junction of Smad7Δ. (A) Mouse embryonic fibroblasts and (B) Human Osteosarcoma cell line HOS. Red arrows indicate the split tags. Since our RNA-seq sequencing depth is too large to be displayed completely, we have truncated the browser view.
(PPT)

S2 Fig. Human SMAD7Δ and mouse Smad7Δ are conserved. (A) Nucleotide sequence spanning the alternative splice site of the human SMAD7 gene. Blue nucleotides are splicing sites. Red and blue nucleotides are skipped in SMAD7Δ. (B) Protein sequence spanning the alternative splice site of human Smad7 gene. Black amino acids are in SMAD7 while red amino acids are in SMAD7Δ. *, stop codon.
(PPT)

Acknowledgments

The authors thank the Yuspa lab for discussions and criticism.

Author Contributions

Conceptualization: AS SHY.

Formal analysis: AS YY SM YH JH.

Funding acquisition: SHY.

Investigation: AS YY SM YH ML CC JH.

Methodology: AS CC JH SHY.

Project administration: SHY.

Resources: AS ML VS C JH.

Supervision: SHY.

Visualization: AS SHY.

Writing - original draft: AS JH SHY.

Writing - review & editing: AS JH SHY.

References

1. Schilling SH, Hjelmeland AB, Rich JN, Wang XF. TGF-b: A Multipotential Cytokine,In: Derynck R, Miyazono K, editors. The TGF-b family. Cold Spring Harbor: Cold Spring Harbor Laboratory Press,; 2008. pp. 45–77.
2. Massague J, Seoane J, Wotton D. Smad transcription factors. Genes Dev, 2005; 19: 2783–2810. PMID: 16322555
3. Imamura T, Takase M, Nishihara A, Oeda E, Hanai J, Kawabata M, et al. Smad6 inhibits signalling by the TGF-beta superfamily. Nature. 1997; 389: 622–626. PMID: 9335505
4. Casellas R, Brivanlou AH. Xenopus Smad7 inhibits both the activin and BMP pathways and acts as a neural inducer. Dev Biol. 2005; 198: 1–12.
5. Hayashi H, Abdollah S, Qiu Y, Cai J, Xu YY, Grinnell BW, et al. The MAD-related protein Smad7 associates with the TGFbeta receptor and functions as an antagonist of TGFbeta signaling. Cell. 1997; 89: 1165–1173. PMID: 9215638
6. Hata A, Lagna G, Massague J, and Hemmati-Brivanlou A. Smad6 inhibits BMP/Smad1 signaling by specifically competing with the Smad4 tumor suppressor. Genes Dev. 1998; 12: 186–197. PMID: 9436979
7. Zhang S, Fei T, Zhang L, Zhang R, Chen F, Ning Y, et al. Smad7 antagonizes transforming growth factor beta signaling in the nucleus by interfering with functional Smad-DNA complex formation. Mol Cell Biol. 2007; 27: 4488–4499. PMID: 17438144
8. Bai S, Shi X, Yang X, Cao X. Smad6 as a transcriptional corepressor. J Biol Chem. 2000; 275: 8267–8270. PMID: 10722652
9. Shukla A, Yuspa SH. CLIC4 and Schnurri-2: a dynamic duo in TGF-beta signaling with broader implications in cellular homeostasis and disease. Nucleus. 2010; 1 144–149. PMID: 20617112
10. Littler DR, Harrop SJ, Goodchild SC, Phang JM, Mynott AV, Jiang L, et al. The enigma of the CLIC proteins: Ion channels redox proteins enzymes scaffolding proteins? FEBS Lett. 2010; 584: 2093–2101. doi: 10.1016/j.febslet.2010.01.027 PMID: 20085760
11. Littler DR, Harrop SJ, Brown LJ, Pankhurst GJ, Mynott AV, Luciani P, et al. Comparison of vertebrate and invertebrate CLIC proteins: the crystal structures of Caenorhabditis elegans EXC-4 and Drosophila melanogaster DmCLIC. Proteins. 2008; 71: 364–378. PMID: 17985355
12. Littler DR, Assaad NN, Harrop SJ, Brown LJ, Pankhurst GJ, Luciani P, et al. Crystal structure of the soluble form of the redox-regulated chloride ion channel protein CLIC4. FEBS J. 2005; 272: 4996–5007. PMID: 16176272
13. Shukla A, Malik M, Cataisson C, Ho Y, Friesen T, Suh KS, et al. TGF-beta signalling is regulated by Schnurri-2-dependent nuclear translocation of CLIC4 and consequent stabilization of phospho-Smad2 and 3. Nat Cell Biol. 2009; 11: 777–784. doi: 10.1038/ncb1885 PMID: 19448624
14. Suh KS, Mutoh M, Nagashima K, Fernandez-Salas E, Edwards LE, Hayes DD, et al. The organellular chloride channel protein CLIC4/mtCLIC translocates to the nucleus in response to cellular stress and accelerates apoptosis. J Biol Chem. 2004; 279: 4632–4641. PMID: 14610078
15. Padmakumar VC, Speer K, Pal-Ghosh S, Masiuk KE, Ryscavage A, Dengler SL, et al. Spontaneous skin erosions and reduced skin and corneal wound healing characterize CLIC4(NULL) mice. Am J Pathol. 2012; 181: 74–84. doi: 10.1016/j.ajpath.2012.03.025 PMID: 22613027
16. Suh KS, Malik M, Shukla A, Ryscavage A, Wright L, Jividen K, et al. CLIC4 is a tumor suppressor for cutaneous squamous cell cancer. Carcinogenesis. 2012; 33: 986–995. doi: 10.1093/carcin/bgs115 PMID: 22387366
17. Shukla A, Edwards R, Yang Y, Hahn A, Folkers K, Ding J, et al. CLIC4 regulates TGF-beta-dependent myofibroblast differentiation to produce a cancer stroma. Oncogene. 2014; 33: 842–850. doi: 10.1038/onc.2013.18 PMID: 23416981
18. Shipley GD, Pittelkow MR, Wille JJ Jr, Scott RE, Moses HL. Reversible inhibition of normal human prokeratinocyte proliferation by type beta transforming growth factor-growth inhibitor in serum-free medium. Cancer Res. 1986; 46:2068–2071. PMID: 2418960
19. Moses HL. TGF-beta regulation of epithelial cell proliferation. Mol Reprod Dev. 1992; 32:179–184. PMID: 1637556
20. Trapnell C, Pachter L, and Salzberg SL. TopHat: discovering splice junctions with RNA-Seq. Bioinformatics. 2009; 25: 1105–1111. doi: 10.1093/bioinformatics/btp120 PMID: 19289445
21. Wang XJ. Role of TGFb Signaling in Skin Carcinogenesis. Microsc Res Tech. 2001; 52:420–429. PMID: 11170301

22. Mochizuki T, Miyazaki H, Hara T, Furuya T, Imamura T, Watabe T, et al. Roles for the MH2 domain of Smad7 in the specific inhibition of transforming growth factor-beta superfamily signaling. J Biol Chem. 2004; 279: 31568–31574. PMID: 15148321

23. Hanyu A, Ishidou Y, Ebisawa T, Shimanuki T, Imamura T, Miyazono K. The N domain of Smad7 is essential for specific inhibition of transforming growth factor-beta signaling. J Cell Biol. 2001; 155: 1017–1027. PMID: 11739411

24. Hallgren O, Malmstrom J, Malmstrom L, Andersson-Sjoland A, Wildt M, Tufvesson E. Splicosomal and serine and arginine-rich splicing factors as targets for TGF-β. Fibrogenesis Tissue Repair. 2012; 5:1–13.

25. Wendt MK, Taylor MA, Schiemann BJ, Sossey-Alaoui K, Schiemann WP. Fibroblast growth factor receptor splice variants are stable markers of oncogneic transforming growth factor b1 signaling in metastatic breast cancer. Breast Cancer Res. 2014; 16:1–15.

26. Ueberham U, Lange P, Ueberham E, Bruckner MK, Hartlage-Rubsamen M, Pannicke T, et al. Smad2 isoforms are differentially expressed during mouse brain development and aging. Int J Dev Neurosci. 2009; 27: 501–510. doi: 10.1016/j.ijdevneu.2009.04.001 PMID: 19375497

27. Jiang Y, Liang H, Guo W, Kottickal LV, Nagarajan L. Differential expression of a novel C-terminally truncated splice form of SMAD5 in hematopoietic stem cells and leukemia. Blood. 2000; 95: 3945–3950. PMID: 10845932

28. Konrad L, Scheiber JA, Bergmann M, Eickelberg O, Hofmann R. Identification of a new human Smad6 splice variant. Andrologia. 2008; 40: 358–363. doi: 10.1111/j.1439-0272.2008.00869.x PMID: 19032685

29. Nishita M, Ueno N, Shibuya H. Smad8B a Smad8 splice variant lacking the SSXS site that inhibits Smad8-mediated signalling. Genes Cells. 1999; 4: 583–591. PMID: 10583507

30. He W, Cao T, Smith DA, Myers TE, Wang XJ. Smads mediate signaling of the TGFbeta superfamily in normal keratinocytes but are lost during skin chemical carcinogenesis. Oncogene. 2001; 20: 471–483. PMID: 11313978

31. Bornstein S, Hoot K, Han GW, Lu SL, Wang XJ. Distinct roles of individual Smads in skin carcinogenesis. Mol Carcinog. 2007; 46: 660–664. PMID: 17477360

32. Stolfi C, Marafini I, De S V Pallone F, Monteleone G. The dual role of Smad7 in the control of cancer growth and metastasis. Int J Mol Sci. 2013; 14: 23774–23790. doi: 10.3390/ijms141223774 PMID: 24317436

33. Liu X, Lee J, Cooley M, Bhogte E, Hartley S, Glick A. Smad7 but not Smad6 cooperates with oncogenic ras to cause malignant conversion in a mouse model for squamous cell carcinoma. Cancer Res. 2003; 63: 7760–7768. PMID: 14633701

34. Akhurst RJ, Hata A. Targeting the TGFbeta signalling pathway in disease. Nat Rev Drug Discov. 2012; 11: 790–811. doi: 10.1038/nrd3810 PMID: 23000686

35. Lichti U, Anders J, and Yuspa SH. Isolation and short-term culture of primary keratinocytes hair follicle populations and dermal cells from newborn mice and keratinocytes from adult mice for in vitro analysis and for grafting to immunodeficient mice. Nat Protoc. 2008; 3: 799–810. doi: 10.1038/nprot.2008.50 PMID: 18451788

36. Zhang X, He Y, Lee KH, Dubois W, Li Z, Wu X, et al. Rap2b a novel p53 target regulates p53-mediated pro-survival function. Cell Cycle. 2013; 12: 1279–1291. doi: 10.4161/cc.24364 PMID: 23535297
